# Supplementary material for: Randomized Controlled Trial of Two Timepoints for Introduction of Standardized Complementary Food in Preterm Infants
Source: Nutrients. 2022 Feb 7;14(3):697. doi: 10.3390/nu14030697 (PMC8839701; doi:10.3390/nu14030697)
Supplement: Supplementary file 1 [file nutrients-14-00697-s001.zip › nutrients-1557299-supplementary.pdf]

Supplementary Materials:
Table S1 : Feeding Boxes

**Starterbox: Scoop familiarization phase (7 products)**<br>Vegetables: (4 products)<br>• parsnip<br>• carrot<br>• carrot and potato<br>• mixed buttered vegetables - carrots, potatoes, peas, corn<br>Fruits: (3 products)<br>• pear<br>• apple<br>• banana-pear
**complementary food box-1: from 5-12 months corrected from term (10 products)**<br>Meat from 5 months on (3 products)<br>• parsnip with potato and veal<br>• vegetable risotto with turkey<br>• carrots with potato and beef<br>Fruits from 5 months on (4 products)<br>• pear-apple<br>• apple-peach<br>• pear-mango<br>• banana-cherry<br>Fruits with cereals from 5 months on (2 products)<br>• apple-grape with rice<br>• banana-peach with rice<br>Cereals from 5 months on (1 product)<br>• semolina porridge
**complementary food box-2: From 6-12 months corrected from term (9 products)**<br>Meat from 6 months on (3 products)<br>• pasta with ham and cream vegetables<br>• buttered vegetables with potato and chicken<br>• spaghetti with cream vegetables and turkey<br>Fruits from 6 months on (2 products)<br>• peach-passion fruit<br>• fruit smoothie - apple, banana, peach, pear<br>Fruits and cereals from 6 months on (3 products)<br>• whole grain fruit-cereal<br>• banana-apple with rusk<br>• apple-banana with whole grain cereal<br>Milk pudding from 6 months on (1 product)<br>• milk pulp with biscuit
**complementary food box-3: From 7/8- 12 months corrected from term (13 products)**<br>Meat from 8 months on (3 products)<br>• couscous with vegetables and chicken<br>• vegetables with rice and turkey<br>• spaghetti bolognese<br>Fruits from 7 months on (3 products)

• peach-plum<br>• apple-plum<br>• fruit smoothie - apple, red fruits, and banana<br>Fruits with yoghurt from 7 months on (3 products)<br>• banana-pear with yoghurt<br>• peach-apricot with yoghurt<br>• yogurt with blueberry<br>Fruits and cereals and cereal bars from 8 months on (3 products)<br>• bear biscuits<br>• yoghurt-cereal with banana-cherry<br>• apple with peach and biscuit<br>Night-mush from 8 months on (1 product)<br>• porridge with apple-pear-banana-yoghurt
**complementary food box-4: from 10-12 months corrected for term (6 products)**<br>Meat and fish from 10 months on (4 products)<br>• spaghetti with tomato and mozzarella<br>• cream vegetables with rice and turkey<br>• pasta in broccoli-cream<br>• baby paella with Alaska salmon and turkey<br>Yoghurt and pudding from 10 months on (2 products)<br>• strawberry yoghurt<br>• chocolate pudding

Table S2 : Anthropometry of the study population during the first year of life: weight, height, head circumference, and corresponding z-scores.

	Early Group (*n* = 83)	Late Group (*n* = 83)	p
**weight**	..	..	
birthweight in g	941 (±253)	932 (±256)	0.73
weight at expected term ±14 days	3249 (±444)	3293 (±467)	0.30
weight with 6 weeks corrected age	4654 (±662)	4594 (±616)	0.43
weight with 3 months corrected age	5811 (±816)	5782 (±739)	0.84
weight with 6 months corrected age	7351 (±1003)	7160 (±868)	0.12
weight with 12 month corrected age	9363 (±1375)	9115 (±1166)	0.15
**z-scores weight**	..	..	..
z-score birthweight	-0.1 (±0.77)	-0.13 (±0.79)	0.64
z-score Weight at expected term±14 days	-0.73 (±0.84)	-0.54 (±0.84)	0.12
z-score Weight with 6 weeks corrected age	-0.47 (±1.01)	-0.44 (±1)	0.15
z-score Weight with 3 months corrected age	-0.67 (±1.14)	-0.64 (±0.95)	0.17
z-score Weight with 6 months corrected age	-0.49 (±1.2)	-0.56 (±1.04)	0.03
z-score weight with 12 month corrected age	-0.18 (±1.31)	-0.3 (±1.13)	0.06
**height**	..	..	..
birthheight in cm	34.8 (±3.1)	34.9 (±3.6)	0.91
height in cm at expected term ±14 days	49.1 (±2.5)	49.2 (±2.6)	0.6
height in cm with 6 weeks corrected age	55.1 (±2.5)	54.6 (±2.4)	0.26
height in cm with 3 months corrected age	60.4 (±2.6)	60.3 (±2.6)	0.82
height in cm with 6 months corrected age	67 (±2.8)	66.3 (±2.7)	0.23
height in cm with 12 months corrected age	74.7 (±2.7)	74.4 (±2.8)	0.73
**z-scores height**	..	..	..
z-score birthheight	-0.01 (±1.02)	0.02 (±1.08)	0.9
z-score height at expected term±14 days	-0.93 (±0.92)	-0.77 (±1.13)	0.43
z-score height with 6 weeks corrected age	-0.59 (±1.13)	-0.66 (±1.2)	0.31
z-score height with 3 months corrected age	-0.34 (±1.12)	-0.36 (±1.11)	0.23
z-score height with 6 months corrected age	0 (±1.22)	-0.1 (±1.16)	0.24
z-score height with 12 months corrected age	-0.31 (±1.05)	-0.3 (±1.12)	0.77
**head circumference**	..	..	..
head circumference in cm at birth	24.8 (±2.2)	24.8 (±2.31)	0.83
head circumference in cm at expected term±14 days	34.6 (±1.6)	34.9 (±1.59)	0.19
head circumference in cm with 6 weeks corrected age	37.7 (±1.5)	37.9 (±1.47)	0.65
head circumference in cm with 3 months corrected age	40 (±1.5)	40 (±1.39)	0.91
head circumference in cm with 6 months corrected age	42.6 (±1.5)	42.6 (±1.54)	0.70
head circumference in cm with 12 months corrected age	45.4 (±1.8)	45.5 (±1.6)	0.62
**z-score head circumference**	..	..	..
z-score head circumference at birth	0.17 (±0.93)	0.15 (±0.93)	0.75
z-score head circumference at expected term ±14 days	-0.34 (±0.98)	-0.01 (±1.03)	0.07
z-score head circumference with 6 weeks corrected age	-0.26 (±1.12)	0.08 (±1.28)	0.48
z-score head circumference with 3 months corrected age	-0.25 (±1.2)	-0.17 (±1.2)	0.14
z-score head circumference with 6 months corrected age	-0.27 (±1.21)	-0.11 (±1.22)	0.38
z-score head circumference with 12 month corrected age	-0.23 (±1.42)	-0.08 (±1.22)	0.25
**BMI**	..	..	..
BMI at birth	7.58 (±1.08)	7.5 (±0.97)	0.63
BMI at expected term ±14 days	13.47 (±1.16)	13.56 (±1.19)	0.27
BMI with 6 weeks corrected age	15.28 (±1.47)	15.33 (±1.62)	0.88
BMI with 3 months corrected age	15.91 (±1.58)	15.89 (±1.52)	0.95
BMI with 6 months corrected age	16.35 (±1.71)	16.25 (±1.64)	0.35
BMI with 12 months corrected age	16.72 (±1.94)	16.44 (±1.83)	0.14
**z-score BMI**			

z-score BMI with 6 weeks corrected age	-0.22 (±1.05)	-0.12 (±1.12)	0.37
z-score BMI with 3 months corrected age	-0.64 (±1.15)	-0.58 (±1.02)	0.77
z-score BMI with 6 months corrected age	-0.66 (±1.23)	-0.67 (±1.15)	0.74
z-score BMI with 12 months corrected age	-0.02 (±1.4)	-0.18 (±1.39)	0.35

p-values were calculated from mixed models adjusted for sex and gestational age, from Visit 1 additionally adjusted for nutrition at discharge (breastfed, formula, mixed), and from Visit 2 additionally adjusted for the respective baseline value. Data are given in mean (SD); BMI=Body mass index

Table S3 : Assessment of interaction effects between nutrition at discharge and treatment group.

Outcome	Predictor	Estimate	Low	Up	p	p treatment	p interaction
Height	Breastmilk feeding	74.313	73.085	75.542	<0.0001	..	..
Height	Formula feeding	73.920	72.818	75.022	<0.0001	..	..
Height	Mixed feeding	73.576	72.656	74.495	<0.0001	..	..
Height	Male sex	0.974	0.192	1.756	0.015	..	..
Height	GA in days (mean centered)	-0.024	-0.052	0.005	0.1014	..	..
Height	Height at Visit 1 (mean centered)	0.505	0.346	0.663	<0.0001	..	..
Height	Breastmilk feeding:Early group	-0.165	-1.689	1.360	0.8311		
Height	Formula feeding:Early group	0.142	-1.275	1.560	0.8426	0.9425	0.873
Height	Mixed feeding:Early Group	0.375	-0.978	1.727	0.5842		
Weight	Breastmilk feeding	9125.995	8555.656	9696.334	<0.0001	..	..
Weight	Formula feeding	9005.821	8483.124	9528.518	<0.0001	..	..
Weight	Mixed feeding	9296.614	8857.435	9735.793	<0.0001	..	..
Weight	Male sex	-168.651	-538.467	201.165	0.369	..	..
Weight	GA in days (mean centered)	-2.104	-15.745	11.537	0.7606	..	..
Weight	Height at Visit 1 (mean centered)	1.423	0.998	1.848	<0.0001	..	..
Weight	Breastmilk feeding: Early group	598.784	-112.452	1310.020	0.0982		
Weight	Formula feeding: Early group	373.319	-299.007	1045.645	0.274	0.2716	0.39
Weight	Mixed feeding: Early Group	-53.531	-697.118	590.057	0.8694		
Head-CF	Breastmilk feeding	45.397	44.717	46.077	<0.0001	..	..
Head-CF	Formula feeding	44.827	44.228	45.426	<0.0001	..	..
Head-CF	Mixed feeding	45.463	44.964	45.961	<0.0001	..	..
Head-CF	Male sex	0.048	-0.373	0.469	0.8221	..	..
Head-CF	GA in days (mean centered)	0.010	-0.006	0.025	0.2322	..	..
Head-CF	Height at Visit 1 (mean centered)	0.589	0.445	0.732	<0.0001	..	..
Head-CF	Breastmilk feeding: Early group	0.061	-0.788	0.909	0.8876		
Head-CF	Formula feeding: Early group	0.073	-0.706	0.853	0.853	0.9597	0.9689
Head-CF	Mixed feeding: Early Group	0.186	-0.553	0.924	0.6193		
BMI	Breastmilk feeding	16.453	15.576	17.331	<0.0001	..	..
BMI	Formula feeding	16.491	15.706	17.277	<0.0001	..	..
BMI	Mixed feeding	17.053	16.397	17.710	<0.0001	..	..
BMI	Male sex	-0.705	-1.269	-0.141	0.0147	..	..
BMI	GA in days (mean centered)	0.009	-0.011	0.030	0.3694	..	..
BMI	Height at Visit 1 (mean centered)	0.685	0.445	0.925	<0.0001	..	..
BMI	Breastmilk feeding: Early group	1.040	-0.045	2.125	0.0601		
BMI	Formula feeding: Early group	0.522	-0.488	1.532	0.3082	0.2026	0.2884
BMI	Mixed feeding: Early Group	-0.109	-1.074	0.856	0.8237		

Estimates were calculated from mixed models explaining the respective outcome at Visit 5 (12 months of age corrected for term) through study group (early vs. late introduction of complementary feeding), the baseline body height obtained at Visit 1 (at term), nutrition at discharge (three categories breastmilk, formula, mixed feeding), gestational age at birth, and sex. A random intercept was included to account for possible correlation between siblings of multiple births. Gestational age and height at Visit 1 were mean centered to facilitate the interpretation of estimated model coefficients. Thus, estimates for breastmilk feeding, formula feeding, and mixed correspond to the estimated mean outcome for patients of female sex in the late group with an average gestational age and average height at Visit 1. Interaction terms are denoted by a colon. The estimates for interaction terms correspond to the estimated difference between early and late introduction of solids in the three different types of nutrition at discharge. Estimates are supported by lower and upper bounds of 95% confidence intervals (columns “Low” and “up”). The p values in the column “p treatment” refer to the null hypothesis that there is no effect of early versus late treatment group in any nutrition at discharge group. The p values in the column “p interaction” refer to the null hypothesis that there is no interaction (i.e. that the difference between early and late group is the same for all nutrition at discharge groups). GA=gestational age, BM=Breastmilk, Head-CF=Head-Circumference, BMI=Body mass index
